# Subphrenic abscess secondary to cervical abscess and fasciitis from dental focus: case report

**DOI:** 10.1186/s13256-019-2036-5

**Published:** 2019-04-28

**Authors:** Caroline Petersen da Costa Ferreira, Marianne Yumi Nakai, Caroline Schmiele Namur, Lucas Ribeiro Tenório, Antonio José Gonçalves

**Affiliations:** 1 Emergence Department, Irmandade da Santa Casa de Misericórdia de São Paulo, São Paulo, Brazil; 2 Head & Neck Surgery Discipline, Surgery Department, Irmandade da Santa Casa de Misericórdia de São Paulo, São Paulo, Brazil; 3 Faculty of Medical Sciences of Santa Casa de São Paulo, São Paulo, Brazil

**Keywords:** Cervical fasciitis, Mediastinitis, Odontogenic abscess

## Abstract

**Background:**

Cervical fasciitis is a group of severe infections with high morbimortality. Reports in the literature of patients with cases evolving with mediastinal dissemination of deep cervical abscess are common. However, cases of abdominal dissemination by contiguity are much rarer.

**Case presentation:**

A 34-year-old Caucasian man presented to the emergency department with a 15-day history of left neck edema, local pain, and fever. Seventeen days prior to presentation, he had undergone odontogenic surgical treatment in a dental clinic. Laboratory examinations did not show meaningful changes. He underwent computed tomography of the neck, thorax, and abdomen, which showed evidence of left collection affecting the retromandibular, submandibular, parapharyngeal, vascular, and mediastinal spaces, bilateral pleural effusion, right subphrenic collection and a small amount of liquids between intestinal loops. A cervical, thoracic, and abdominal surgical approach at the same surgery was indicated for odontogenic cervical abscess, descending necrotizing mediastinitis, and subphrenic abscess. The patient remained in the intensive care unit for three days, and he was discharged on the 22nd day after surgery with no drains and no tracheostomy. His outpatient discharge occurred after 6 months with no sequelae.

**Conclusions:**

Aggressive surgical treatment associated with antibiotic therapy has been shown to be effective for improving the clinical course of cervical fasciitis. Despite the extension of the infection in our patient, a surgical approach of all infectious focus associated with a broad-spectrum antibiotic therapy led to a good clinical evolution and has significant implications for aggressive treatment.

## Background

Cervical fasciitis (CF) is a group of bacterial infections, usually of odontogenic or amygdala origin, characterized by necrosis of the fascia and subcutaneous cellular tissue of the neck [1, 2]. It is a rare disease with high morbimortality rates. It may evolve with mediastinitis, especially in patients with comorbidities and immunosuppression [3, 4]. Approximately 46% of the patients with CF arising with mediastinitis show at least one of the risk factors: diabetes mellitus, atherosclerosis, alcohol abuse, kidney failure, neoplasia, intravenous drug abuse, or postpartum status [5–7]. Mediastinitis occurs through infection propagation (fasciitis) in the deep spaces of the neck that continue with mediastinal fascia and the pericardium. Usually, the clinical scenario in this case is severe sepsis or septic shock [8, 9].

CF diagnosis is based on a combination of clinical presentation and imaging. It usually involves polymicrobial infection, both aerobic and anaerobic [2]. Early diagnosis and precise treatment of this pathology in its early stage (before clinical signs of deterioration) are critical in the prognosis of these patients [10].

This report describes a case of a rare disease with an unusual presentation. It is worth highlighting the fact that despite the rarity, it is a serious condition with high potential for morbidity and mortality. Often its diagnosis and treatment are delayed because of the difficulty in identifying the symptoms and the severity of the case. Several chest complications of deep cervical abscess [7–10] have been reported in the literature, but abdominal involvement is uncommon. Some cases of liver abscess through hematogenic dissemination [11–13] from CF have also been reported. However, abdominal dissemination by contiguity (such as the case we report herein) is much rarer. It occurred in one case of retroperitoneal localization [14] and another one with abdominoperineal extension [15].

With this information in mind, it is important to understand that it is a unique presentation of a severe condition that usually presents with a fast progression to sepsis with a high mortality rate. The identification of this kind of dissemination can prevent an unfavorable outcome by giving the physician’s team the whole picture of the case, allowing them to intervene at all sites of the infection.

## Case presentation

A 34-year-old Caucasian man presented to the emergency department with a 15-day history of left neck edema, local pain, and fever. He was in good general condition, awake, lucid, oriented but discretely dyspneic. He had trismus (70%); a painful, warm, red, hard left neck bulging of 10 cm in length without signs of fluctuation; fever (temperature 38.8 °C); ventilator-dependent pain in the right hemithorax; respiratory rate of 23 breaths/min; pulse rate of 100 beats/min, and abdominal discomfort in the right hypochondrium. His blood pressure was within normal range at 110/70 mmHg. He had no alterations in cardiac auscultation and a reduced vesicular murmur at the right hemithorax. His abdomen was discretely distended, and he had considerable pain in the right hypochondrium. His Murphy’s sign was negative, he had no sign of peritonitis. He did not present with any neurological symptoms.

The patient and his wife and children lived in a small house with a monthly income of approximately 410 dollars. There was no record of a permanent job, but he formerly worked in construction as a bricklayer. He had no significant past medical history, but he reported daily smoking (20 cigarettes/day) for 20 years and alcohol consumption of 500 ml of distillate drinks on a daily basis for 10 years. He denied illicit drug consumption.

Seventeen days prior to presentation, he had undergone odontogenic surgical treatment in a dental clinic. He received oral amoxicillin 500 mg/8 h two days prior to the dental procedure, and after that, he received it for five more days with dipyrone (1 g/6 h) and nimesulide (100 mg/12 h). After the dental procedure, seven days before his admission to our unit, he went to another emergency room (ER) with the same symptoms, and there he received empirical intravenous antibiotic therapy (ciprofloxacin 400 mg/12 h) for seven days. Realizing that he was not improving, he left the first ER and presented to our hospital.

**Fig. 1:**
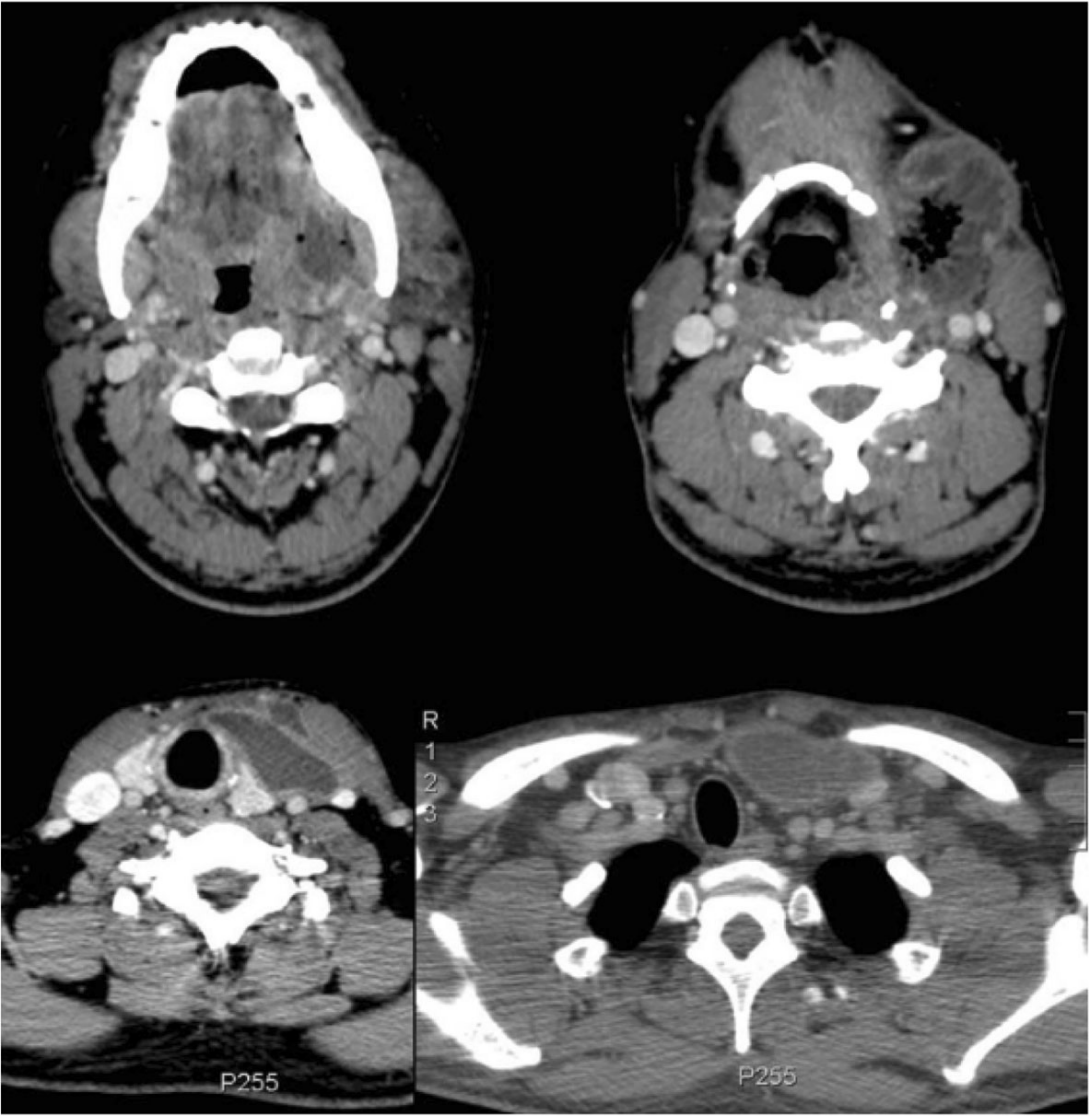
Computed tomography of the neck with evidence of extension of the left collection affecting the retromandibular, submandibular, parapharyngeal, and vascular spaces, as well as the upper mediastinum

**Fig. 2:**
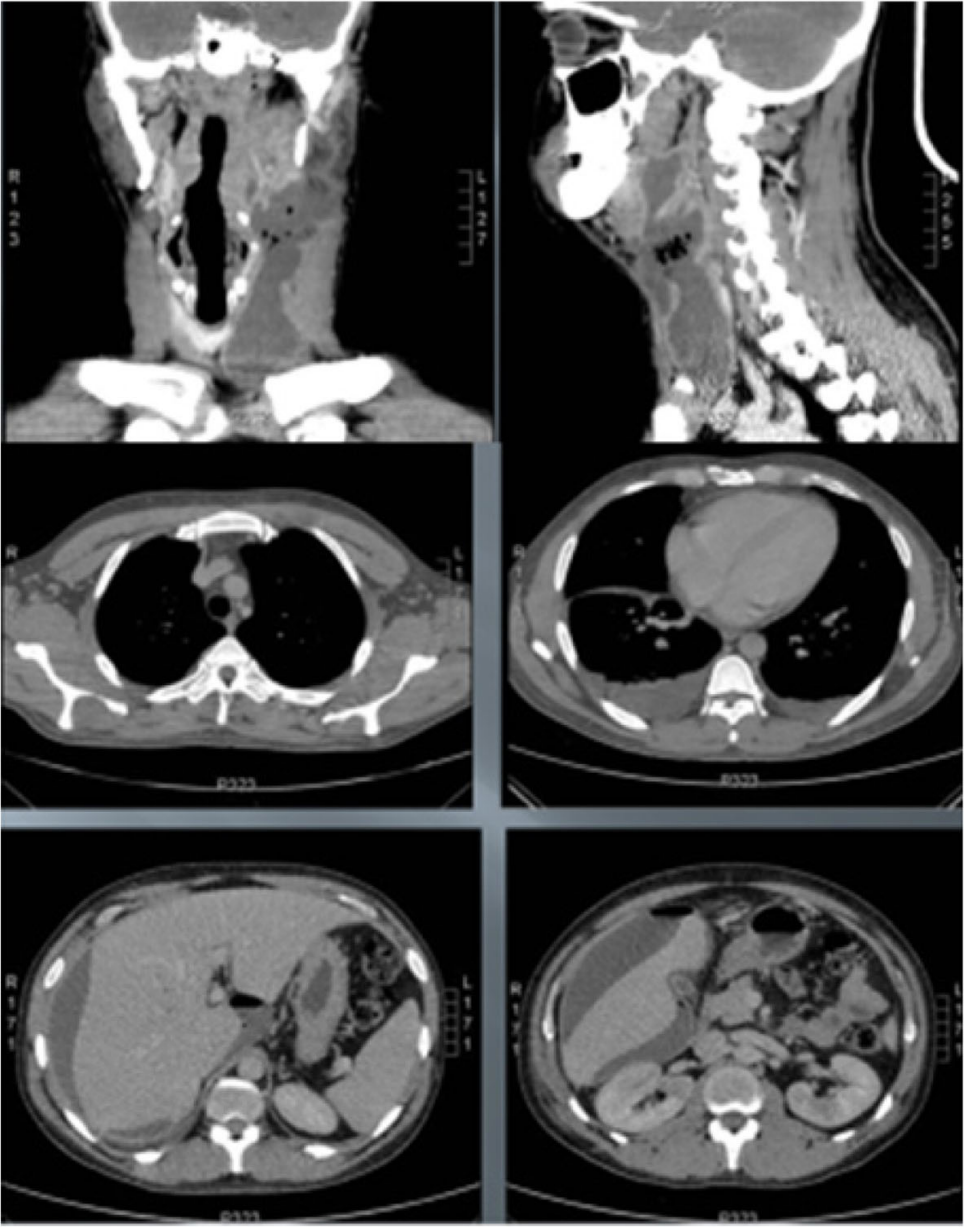
Computed tomography of the neck, thorax, and abdomen showing process extension

Upon his admission to our hospital, we started to manage the sepsis with an intravenous isotonic saline solution and empirical broad-spectrum antibiotics (1000 ml of 0.9% sodium chloride solution + ceftriaxone 1 g/12 h + clindamycin 600 mg/6 h). We planned antibiotic administration for 21 days, and it was given as intravenous dipyrone (1 g/6 h) for fever and tramadol (100 mg/8 h) for pain. Laboratory examinations did not show meaningful changes (Table 1). The patient underwent serological screening to rule out possible causes of immunodeficiency (Table 2), and blood cultures were collected. He underwent cervicothoracoabdominal computed tomography (CT) (Figs. 1 and 2). CT of the neck demonstrated soft-tissue infiltration and edema of the muscular tissue; left retromandibular, submandibular, parapharyngeal, and vascular space collections; and another left upper encapsulated fluid mediastinal collection. CT of the thorax and abdomen demonstrated bilateral pleural effusion, right subphrenic collection, and a small amount of liquid between intestinal loops.

**Table 1:** Laboratory examinations upon admission

Laboratory examinations	Admission values	Reference values
Hemoglobin	9 .7 g/dl	14–18 g/dl [17]
Leukocytes	10,800/μl, no deviations	4,000–11,000/μl [17]
C-reactive protein	4 .4 mg/dl	0 .8 mg/dl or less^a^ [17]
Creatinine	0.6 mg/dl	0.7–1 .5 mg/dl [17]
Urea	17 mg/dl	8–20 mg/dl [17]

^a^C-reactive protein: low risk, < 1.0 mg/L; average risk, 1.0–3.0 mg/L; high risk, > 3.0 mg/L [16]

**Table 2:** Serology

Laboratory examinations	Result
HIV (1 and 2)	Negative
HTLV (1 and 2)	Negative
Hepatitis C serology	Negative
Hepatitis B serology	Negative

*HIV* Human immunodeficiency virus, *HTLV* Human T-cell leukemia-lymphoma virus

**Fig. 3:**
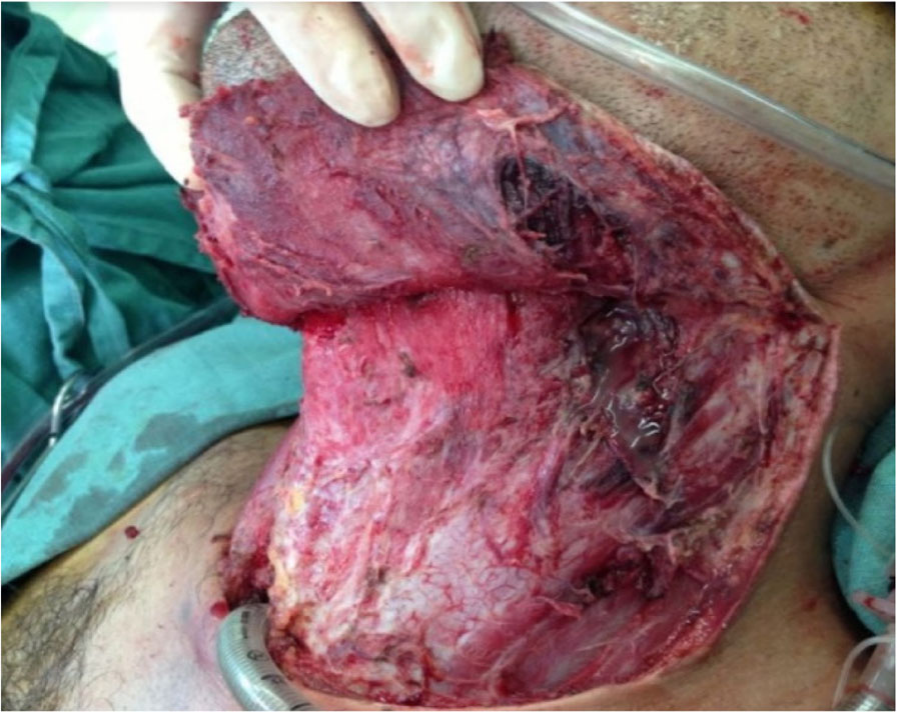
Final aspect of the cervicotomy

**Fig. 4:**
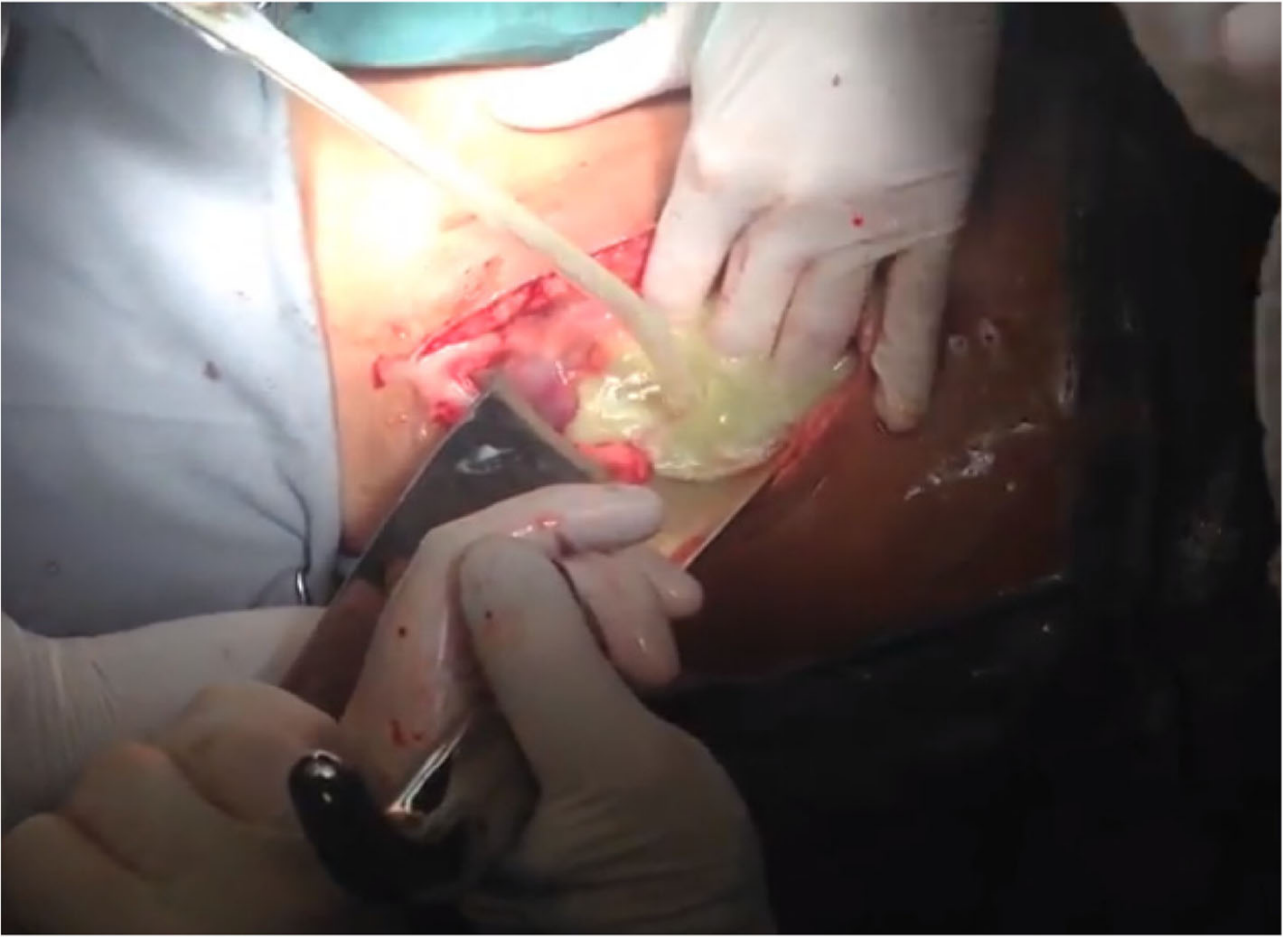
Aspect of secretion after break of the capsule in the subphrenic abscess intraoperatively

**Fig. 5:**
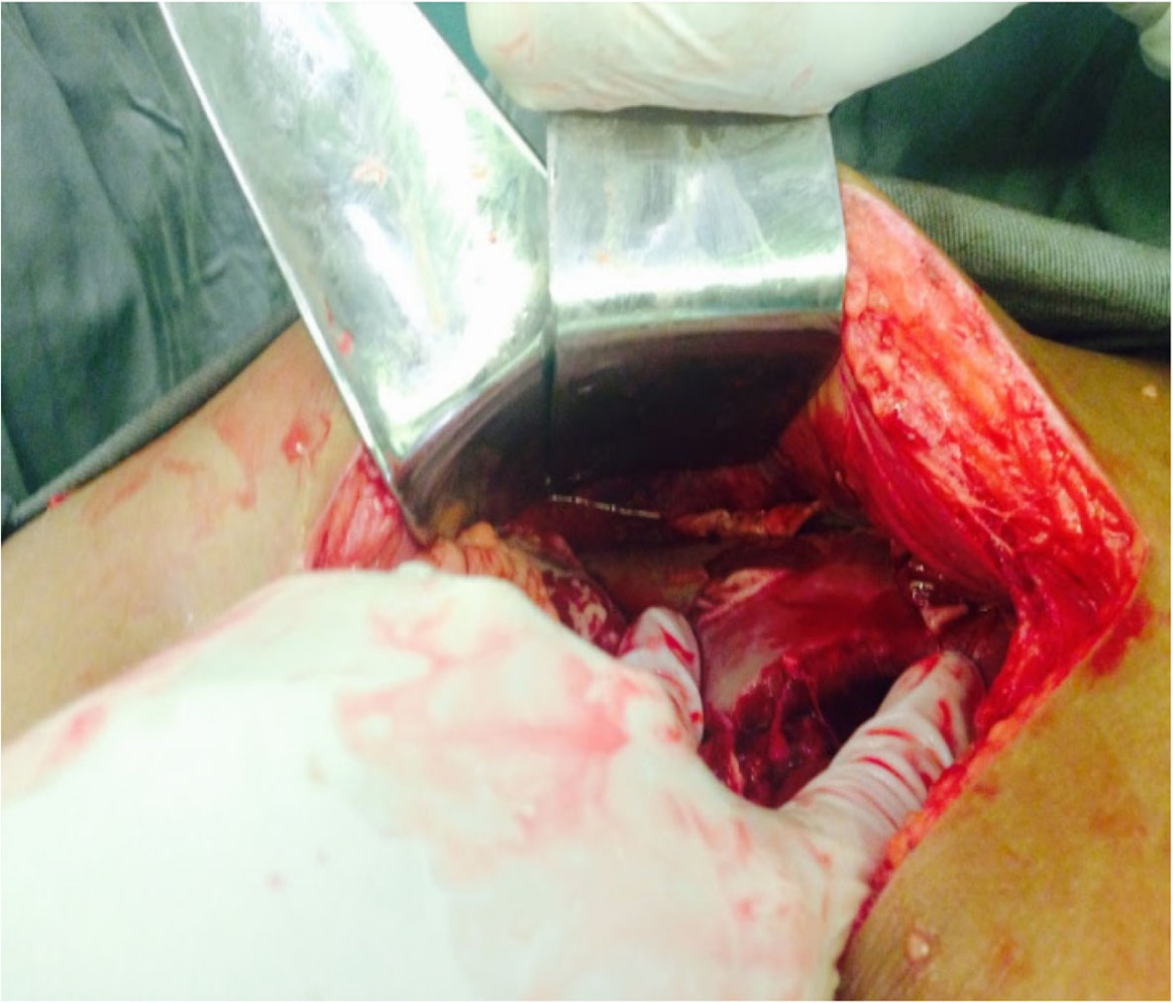
After aspiration of the purulent secretion, a well-formed subphrenic abscess cavity is evidenced by the presence of fibrin

Initially, based on these data, it was concluded that the patient had deep neck space infection (with involvement of the cervical fascia) from a dental focus, descending necrotizing mediastinitis, and subphrenic abscess; therefore, the patient underwent cervical, thoracic, and abdominal aggressive operative drainage. After bronchoscopic intubation without muscle blockers, the surgery was started by left cervicotomy, evidencing an abscess cavity in the submandibular and retromandibular spaces in addition to a small amount of thick liquid in left vascular and retropharyngeal spaces and upper mediastinum. The procedure consisted of extensive debridement and drainage of the cervical and mediastinal collection with tracheostomy to secure the airway (Fig. 3). Drainage tubes were left in place, and samples were taken for culture and an antibiogram. Afterward, a right posterolateral thoracotomy was performed, evidencing serum pleural effusion and mediastinal and pericardial thickening. Mediastinal and pleural drainage was done via large-bore chest tubes. An abdominal approach through median supraumbilical laparotomy revealed a right subphrenic abscess. After drainage of 2000 ml of purulent secretion (Figs. 4 and 5), drainage tubes were also left in place, and samples were taken.

The following day, the patient had septic shock and needed vasoactive drugs. The antibiotic treatment was changed to intravenous piperacillin and tazobactam 4 g/6 h and vancomycin 1 g/12 h.

The bacteriologic results from materials obtained from the abdomen revealed only *Candida albicans.* Aerobic and anaerobic liquid cultures were collected with sterile syringes in the surgical field and were stored in dry tubes for aerobic pathogens and in appropriate liquid contents for anaerobes. We don't have information about how was made the transportation of the tubes. There was no growth in the neck, pleura, and mediastinum material. The blood culture was also negative. Gram staining did not detect any bacteria. These were probably due to the patient’s previous use of antibiotics. Fluconazole (400 mg/12 h) was added to the treatment.

After three days in the intensive care unit, the patient was hemodynamically stable with apparent improvement, and he was transferred to the head and neck ward. He had 21 days of antibiotic treatment (2 days of ceftriaxone 1 g/12 h + clindamycin 600mg/6 h, 19 days of piperacillin and tazobactam 4 g/ 6 h) and 15 days of antifungal treatment (fluconazole 400 mg/12 h), and then he was discharged from the hospital on the 22nd postoperative day, without drains or tracheostomy. His outpatient discharge occurred six months later, without any sequelae. At his last appointment, the scars had a good aspect, with no keloids or hypertrophy, and he had no complaints about swallowing, breathing, or vocal hoarseness. He was able to get back to his work with no restrictions.

## Discussion

This report describes a case of a young man who, after dental treatment for periodontal infection, had an unfavorable evolution with cervical infection and progression to the thorax and abdomen. He underwent broad-spectrum antibiotic therapy in addition to the general measures for sepsis and aggressive surgical treatment with cervicotomy, thoracotomy, and laparotomy for drainage of all abscess stores. This treatment led to a good evolution and favorable outcome with discharge in a reasonably short time and return to usual activities. The peculiarity and severity of this case is a reminder that treatment of a cervical abscess should include intravenous broad-spectrum antibiotics and surgical drainage of the purulent collections. As highlighted before, this case report describes a rare serious disease with an unusual presentation with a high potential for a rapid course and unfavorable outcome. Most of the time, in unspecialized health units, the diagnosis and treatment can be delayed because of the difficulty in identifying the symptoms and the severity of the case. Despite the scarcity of records regarding the disease and the lack of uniformity of the therapeutic approach in different health units, there is a consensus that aggressive and early surgical treatment gives the patient the best chance of survival [3, 4].

The global literature reports several cases of mediastinal dissemination of deep cervical abscess [7–10]. Although rare, there are some cases of hepatic abscess through hematogenic dissemination [11–13]; however, abdominal dissemination by contiguity is even rarer, and it occurred in only one case of retroperitoneal localization [14] and another with abdominoperineal extension [15].

A good understanding of the cervical fascial anatomy is essential for CF diagnosis and its possible complications, because clinical presentation and dissemination are based on deep cervical spaces. The perivascular space contains the carotid artery, internal jugular vein, and vagus nerve. The prevertebral (or retrovisceral) space is divided into retropharyngeal and danger spaces by the alar fascia. Perivascular and prevertebral spaces are contiguous with the upper mediastinum and pretracheal space with the anterior mediastinum. Infections affecting such spaces show a higher chance of evolving to mediastinitis [17, 18].

Diagnosis may be difficult, especially with the previous use of antibiotics, where symptoms may be masked, and dissociation may be found between the clinical condition and the imaging examination. CT is vital to good therapeutic planning, being the gold standard for this evaluation [19]. The most common pathogens involved in this kind of infection in Brazil are *Staphylococcus aureus* and *Streptococcus viridans*, although most often a polymicrobial flora with anaerobic pathogens from regular oral flora is found [4].

Risk factors associated with a poor prognosis are the following: diabetes mellitus, alcohol abuse, poor oral hygiene, malnutrition, corticotherapy, improper treatment with antibiotics, and time to treatment (time between diagnosis and treatment: 6–18 h: 28.5% of mortality; time between diagnosis and treatment > 18 h: 42% of mortality) [19].

Surgical approach with broad cervicotomy for exploration of deep spaces, washing, and drainage associated with focus-driven antibiotic therapy should be the base of CF treatment [18, 20].

The airway may be a challenge in the initial approach of these patients, who often show trismus, secretion, edema, and extrinsic bulging, which can make the management of  the airway difficult. Techniques such as awake orotracheal intubation under topic anesthesia of the airway, with or without bronchoscopic and awake tracheostomy under local anesthesia are the safest alternatives.

Abdominal dissemination of CF by contiguity is not common. The previous use of antibiotics, among its actions, may contain the infection to a restricted space, increasing the dissection pressure in this compartment and making it easier to propagate it by contiguity, which may be one of the factors that may have contributed to its occurrence in our patient.

In summary, CF remains a life-threatening infection. A deep understanding of the natural history of this infection and the local anatomy can save lives. Early diagnosis and surveillance with contrast-enhanced CT are essential. The extension of surgical drainage should depend on the affected spaces found by CT and intraoperatively.

## Conclusions

Although CF is rare, it has a high rate of morbimortality if diagnosed late, owing to the involvement of deep cervical spaces and predisposition to thoracic, and abdominal dissemination with worse prognosis. Aggressive surgical treatment associated with antibiotic therapy has been shown to be effective in improving the patient’s clinical condition.

This report describes a patient with a 15-day history of infection empirically treated with antibiotics, who showed cervicothoracoabdominal dissemination. Despite the infection extension, a surgical approach of all infectious focus associated with broad-spectrum antibiotic therapy led to a good clinical evolution.

## Abbreviations

CF: Cervical fasciitis
CT: Computed tomography
ER: Emergency room
